# Myths

**Published:** 1883-10

**Authors:** Ausburn Towner


### MYTHS.

AUSBURN TOWNER.

The creation of myths, or imaginary characters or beings, having their foundations in desires of the heart or observations of nature, is one of the most natural of the operations of the human mind. There is an universal element of the intellect, even of the most uncultured, perhaps applying most especially to the most uncultured, that takes delight in this exercise. Some, from this faculty, draw a strong argument in favor of spirituality in opposition to materialism, as if the soul was conscious, involuntarily, of another existence where everything is brighter, better, purer and happier than in this world, and was trying to reach that place, if not in practical experience, at least in dreams. The lowest form of this faculty is seen in the savage, who "sees God in the cloud or hears him in the wind," and in child-life, that is peopled with myths, like Cinderella, Tom Thumb, Puss-in-boots, Hop o' my Thumb, and Jack the Giant Killer; or that evolves from its own inner consciousness, the notion that the raggedest of rag dolls is a real princess, or that the most commonplace and dilapidated toys are priceless treasures. In its highest development it appears in the poet, who makes his myths so real that they cease to be shadowy or immaterial. Oliver Wendell Holmes sets forth this idea upon which I wish to touch with his usual facility and felicity of expression, as follows:

> There breathes no being but has some pretence
> To that fine instinct called poetic sense;
> The rudest savage roaming through the wild,
> The simplest rustic bending o'er his child,
> The infant listening to the warbling bird,
> The mother smiling at its half-formed word;
> The boy uncaged, who tracks the fields at large,
> The girl turned matron to her babe-like charge;
> The freeman, casting with unpurchased hand
> The vote that shakes the turrets of the land;
> The slave, who, slumbering on his rusted chain,
> Dreams of the palm-trees on his burning plain;
> The hot-cheeked reveller, tossing down the wine,
> To join the chorus pealing "Auld Lang Syne;"
> The gentle maid, whose azure eye grows dim,
> While Heaven is listening to her evening hymn;
> The jewelled beauty, when her steps draw near
> The circling dance and dazzling chandelier;
> E'en trembling age, when Spring's renewing air
> Waves the thin ringlets of his silvered hair;—
> All, all are glowing with the inward flame
> Whose wider halo wreaths the poet's name."

Holmes calls this faculty of myth-creating a "poetic sense." It is so, not always in its power to write rhymes, or express itself in rythmic numbers, but in its ability to create images. You may have heard some hard-headed, close-fisted man inveigh strongly against this faculty and allude to those possessing it in large measure as persons who "delve after the unfathomable and reach for the unattainable, but who never pay cash!" It is an even chance that these very same hard-headed ones have an ideal or a myth somewhere that controls their lives and is eating away the better part of their natures. The grasping after money, the listening to the cries of ambition, the yearning after fame, or the burning enthusiasm of the fanatic, are just as much myths to those led in such directions by the faculty of the mind I speak of, as are the visions seen by the poet or dreamed out by the novelist. They differ only in kind, not in quality. Supposed attainment or realization of the hope in the heart only shows how misty and unreal, how uncertain and foundationless, how unsatisfying are all the objects for which man can strive and struggle in this world. When death loosens the grasp of even the greatest and most powerful, how true it is that, after all, he finds nothing there, and the open palms show how empty they have really been. This personal characteristic, strong in some men, weak in others, but belonging to all, makes it less to be wondered at that the larger myths, coming down to us from the ages past, have such a firm hold upon mankind, and, although in many instances dissipated by investigation and the vigorous inquiries of philosophers, still hold place in our literature and the common speech of all races. One hardly knows whether to sympathize with the recent inquirers who have resolved most of what are known as myths into personifications of the various operations of nature by our ancestors of a remote period, or to laugh them down as themselves the more mythical, and believe rather that there was really an Apollo, an Endymion, an Aurora, than that the first stood simply for the sun, the second for the day, and the third for the dawn. It is hard to believe that our stories of Cinderella, Puss-in-boots, the Sleeping Princess, Beauty and the Beast, and the multitude of others like them, are not mere inventions by apt story-tellers, but are myths, growing up centuries and centuries ago, and represent sometimes day and night, sometimes the seasons or the whole year. These are ingenious theories set forth by modern inquirers, and are developed very largely, first, to show that as these tales appear in one form or another in the folk-lore of every race under the sun, mankind must have had one origin, or must have sprung from one source; and, second, to account for the mythology or religion of every character that the world knows or has known, except, perhaps, the Christian. Yet there is danger here, for some one, following out the line of reasoning, has tried to show that Christ himself was a myth of a similar character to these of which I speak. He, they say, stands for the Sun, going in his yearly path through the heavens; his apostles are the twelve months, and his death occurs at the end of what was then known as the Roman year. I do not care to follow out this peculiar sort of mythical ingenuity, but want to give a more striking example of the ease with which a real history can be turned into the line of thought we are considering. It shows upon what unsafe ground all such conjectures are based, and what little reliance can be placed on any reasoning. It concerns Napoleon Bonaparte. Everyone must have read Archbishop Whateley's ingenious book called "Historic Doubts as to Napoleon Bonaparte." In this the witty ecclesiastic very logically demonstrates that no such person could possibly have lived, but that to which I refer demonstrates that Bonaparte was only a Sun-myth. It is a book by a French Abbe, and was evidently written as a satire upon those German inquirers who, as I have said above, have referred back to the original days of the human race and to the personification of operations of nature, the stories that form so large a part of our child-life literature.

In the first place the French Abbe makes the likeness perfect between the name of Napoleon and Apollo, or Apoleon, the god of the sun. On the column of the Place Vendome, Napoleon's name is spelled Nèapolèo. Now the Greek prefix *Ne* is strongly affirmative in its character, so that Ne-apoleo means: This is the very Apollo and no mistake, the genuine, original Jacobs.

The other name, Bonaparte, makes this stronger. The day has two parts, the good, or luminous portion, and the bad or dark. To the sun belongs the good part, and it is quite proper that his surname should therefore be *Bona*, good; *parte*, part. Apollo was born on an island in the Mediterranean, so was Napoleon; Apollo was an Egyptian deity held in high reverence. So we find Napoleon in Egypt, regarded by the inhabitants with veneration and received with homage. The name of Napoleon's mother was Letitia, which signifies joy, and is a personification of the dawn from which the sun springs. The name of Apollo's mother was Latone, which is the Latin form of the same word. Napoleon had three sisters. So did Apollo, whose sisters were the graces. Napoleon had four brothers; Apollo the same, representing the four seasons. Three were kings, reigning respectively: Spring, over the flowers; Summer, over the harvest; Autumn, over the fruits. As there are three seasons, due all to the powerful influence of the sun, so in the sight of Napoleon did his three brothers draw all their authority from him. The fourth brother was an impersonification of Winter. In the decline of Napoleon's power he was given authority over the small principalty of Canion, a name derived from *cani*, or the whitened hairs of old age—true emblem of winter.

Napoleon is said to have had two wives. Classic fable gives the same number to Apollo. These two were the moon and the earth. By one he had no posterity; by the other one son only, the little Horus, representing the fruits of agriculture produced by the earth, fertilized by the sun. The pretended son of the fabulous Napoleon was born on the 20th of March, the season of the spring equinox, when agriculture is assuming its greatest period of activity.

Napoleon is said to have saved France from a devastating scourge, the hydra of the revolution, an incident also in the life of Apollo. Indeed, revolution is derived from the Latin revolve, indicating clearly the coils of the serpent like the Python. Napoleon's sixteen marshals represent the twelve signs of the zodiac and the four cardinal points. The story of his campaign in the north is but a picture of the Sun moving into that region and then being driven back. That's all upon which is founded the fatal march to Moscow and the humbling retreat therefrom. And finally, the sun rises in the east and sets in the western sea. The poets picture him rising out of the waters of the east and setting in the ocean after his twelve hours reign in the sky. Such is the history of Napoleon, coming from his Mediterranean isle, holding the reins of government for twelve years, and finally disappearing in the mysterious region of the great Atlantic.

It will thus be seen how easily, with man's natural love for the mythical, well-authenticated history may be made legendary and full of mystery. It may be that in coming ages, men not being able to understand the greatness of the characters of men like Washington or Lincoln, as we do not understand fully the patriarchal era, may take the two names to stand for ideas or personifications of great events and regard them simply as myths. Indeed, ignorant of whence we come, equally in knowing as to whither we are bound, foregetful of the past, uncertain as to the present and blind as to the future, what is man at the best, but a myth having no knowledge of himself and never understanding those who are the nearest to him? It is, therefore, no wonder, returning to the thought with which I started out, that the most natural operation of the human mind should be the creation out of the shadowy and uncertain realm we occupy, of equally shadowy and uncertain forms or ideas that may be ideals of what we may ourselves desire to be or what we may deem we have been. The world is better for these things, immeasurably better when they are the offspring of a genius, and it is no myth to hope, that upon them mankind will continually rise until a period arrives when no such incentives will be required to hold all on a plane of the highest mental and moral elevation.

